# Comprehensive care lowers multimorbidity, impairment, disability and absenteeism in people with TB

**DOI:** 10.5588/ijtldopen.25.0704

**Published:** 2026-03-13

**Authors:** F. Mavhunga, K. Viney, A. Baddeley, C.M. Halleux, G. Aslanyan

**Affiliations:** 1 Department for HIV, Tuberculosis, Hepatitis and Sexually Transmitted Infections, World Health Organization, Geneva, Switzerland;; 2 UNICEF/UNDP/World Bank/WHO Special Programme for Research and Training in Tropical Diseases (TDR), World Health Organization, Geneva, Switzerland.

**Keywords:** tuberculosis, Kenya, Uganda, Zambia, Zimbabwe, post-TB lung disease, PTLD

## Abstract

A substantial proportion of TB survivors experience poor health, live with comorbidities or experience impairments or disability, even when TB is successfully treated. In this study, implemented in Kenya, Uganda, Zambia and Zimbabwe, this disease burden was addressed through a package of screening, referral and assessment for functional impairments at the start and end of TB treatment. Reductions in symptoms, harmful behaviours, functional impairments and absenteeism were observed. These promising results demonstrate the importance of early intervention. They could be enhanced by implementing this package of interventions in other high TB burden settings and by longer-term follow up.

In the global fight against TB, 83 million lives have been saved since the year 2000.^1^ However, of the estimated 155 million TB survivors (from 1980–2019),^2^ a substantial proportion experience poor health, live with multiple comorbidities, and experience TB-associated impairments or disability.^3–5^ These challenges undermine livelihoods and learning, reduce health-related quality of life, and heighten the risk of TB recurrence and death. The case for providing early, comprehensive care for people with TB – including interventions that address health-related risk factors, comorbidities, impairments and disability – has been strongly advocated.^6–8^ The WHO recently issued a policy brief on TB-associated disability,^9^ emphasizing the need for people-centered, integrated care that includes screening and detection, referral, and coordinated multidisciplinary approaches within and beyond the health sector, including for social protection. In addition, WHO has published a Framework for collaborative action on TB and comorbidities, which aims to support countries in the introduction and scale-up of holistic people-centred services to address TB, comorbidities and health-related risk factors.^10^ WHO also has specific recommendations on assessing for key health-related risk factors and comorbidities among people with TB and providing the appropriate care and management for those diagnosed.^11^ To advance the recommendations in WHO policy documents and national TB strategic plans, implementation research is essential to generate practical knowledge on how to apply them in real-world programmatic settings and to assess their impact on the health and well-being of people with TB.

In 2023, the Kenya, Uganda, Zambia, and Zimbabwe TB Disability Study Group demonstrated the feasibility of conducting screening and referrals for health-related risk factors and co-morbidities as well as functional impairments at the end of TB treatment.^12^ Using standardized screening questions and tools, people with TB (aged 18 years and above) were assessed for a range of comorbidities and health-related risk factors (comprising HIV, diabetes mellitus, hypertension, mental health disorders, the use of tobacco, alcohol and other recreational drugs, silica exposure and undernutrition), functional cardio-pulmonary impairment (using the six-minute walk test [6MWT]) and absenteeism from school or work. People who screened positive were followed up and were referred to appropriate health services. Given that a high prevalence of these conditions was identified in this study, a key operational recommendation was to initiate screening and referrals at the start of TB treatment. The underlying rationale is that ‘acting early’ has the potential to improve overall health outcomes by reducing the burden and potentially, severity, of these conditions by the end of TB treatment. Early detection and management of risk factors, comorbidities and impairments also have the potential to lower healthcare costs by avoiding expensive treatments required for advanced disease and reducing the need for hospitalization and long-term care. In this context, the latest evidence generated by the same study group in this issue of the Journal is encouraging in several ways.^13^ First, all participating countries implemented screening for multiple conditions and impairments both at the start and end of TB treatment, with a median assessment time of approximately 30 minutes, demonstrating feasibility. Second, there were notable reductions in symptoms such as cough and conditions such as probable depression, as well as harmful behaviours, including smoking, excessive alcohol use, and recreational drug use. Importantly, the proportion of participants with impairment or disability – defined as being unable to walk 400 meters in six minutes – decreased from 20% to 4%, showing that this impairment could be minimized. While it was not possible to specifically attribute TB to this impairment, TB is a likely cause given the temporal relationship and given that TB-associated lung disease is being increasingly recognized as a consequence of TB disease.^14^ These proportions are well below what was found in a previous study^12^ conducted in the same settings where assessments were conducted only at the end of TB treatment, without the possibility of early intervention. The package of early screening and linkage to care was associated with significant improvements in health and well-being. Third, the package of early interventions likely resulted in improvements in patients’ overall health, which in turn may have led to a significant drop in absenteeism from school and work, from 73% at treatment initiation to just 10% at treatment completion. Although the direct effects on wages, financial vulnerability, learning and livelihoods were not formally measured, it is reasonable to infer that comprehensive care accelerates improvements in the capacity for regular work and school attendance. Finally, thorough screening and assessment resulted in the identification and referral of new conditions at both the beginning and end of TB treatment, fostering a continuum of care throughout and beyond the TB treatment period, aligned to the person’s health needs. This work now needs to be expanded. More evidence from diverse countries and contexts is required to further inform global and national policies. Inclusion of children would be desirable, with screening tailored more specifically to their health needs, using tools that are validated in this population. This highlights an area where further research and guidance is needed.

Within health facilities, referrals for disability and silica exposure were identified as specific gaps. This highlights the need to develop and scale up community-based and culturally appropriate point-of-care physiotherapy for people with impairments and to implement stronger measures to reduce silica exposure. In countries such as Zambia and Zimbabwe, where mining-related silica exposure is commonly reported, closer engagement with mining authorities and affected communities is essential to identify sources of silica exposure, establish protective measures and offer occupational health services, including TB screening and prevention, which is currently recommended by WHO.^15^ Additionally, longitudinal studies are warranted to assess the long-term impact of early interventions on recurrent TB and overall survival.^6^

## CONCLUSION

The ongoing work led by the National TB Programmes in Kenya, Uganda, Zambia, and Zimbabwe to enhance comprehensive, people-centered care for people with TB is highly commendable. This approach moves TB treatment beyond a purely medical focus, toward holistic care. It also underscores the longer-term benefits of timely and feasible screening for comorbidities, health related risk factors and impairments – some of which persist beyond the course of TB treatment. The encouraging results observed so far reinforce the imperative to scale and sustain these transformative initiatives.
